# Effect of Methionine Diet on Metabolic and Histopathological Changes of Rat Hippocampus

**DOI:** 10.3390/ijms20246234

**Published:** 2019-12-10

**Authors:** Maria Kovalska, Petra Hnilicova, Dagmar Kalenska, Barbara Tothova, Marian Adamkov, Jan Lehotsky

**Affiliations:** 1 Department of Histology and Embryology, Jessenius Faculty of Medicine, Comenius University in Bratislava, 03601 Martin, Slovakia; maria.kovalska@uniba.sk (M.K.); marian.adamkov@uniba.sk (M.A.); 2 Department of Neuroscience, Biomedical Center Martin, Jessenius Faculty of Medicine, Comenius University in Bratislava, 03601 Martin, Slovakia; petra.hnilicova@uniba.sk; 3 Department of Anatomy, Jessenius Faculty of Medicine, Comenius University in Bratislava, 03601 Martin, Slovakia; dagmar.kalenska@uniba.sk; 4 Department of Medical Biochemistry, Jessenius Faculty of Medicine, Comenius University in Bratislava, 03601 Martin, Slovakia; 5 Department of Molecular Medicine, Biomedical Center Martin, Jessenius Faculty of Medicine, Comenius University in Bratislava, 03601 Martin, Slovakia; barbara.tothova@uniba.sk

**Keywords:** hippocampus, hyperhomocysteinemia, neurodegeneration, methionine diet, 1H MRS

## Abstract

Hyperhomocysteinemia (hHcy) is regarded as an independent and strong risk factor for cerebrovascular diseases, stroke, and dementias. The hippocampus has a crucial role in spatial navigation and memory processes and is being constantly studied for neurodegenerative disorders. We used a moderate methionine (Met) diet at a dose of 2 g/kg of animal weight/day in duration of four weeks to induce mild hHcy in adult male Wistar rats. A novel approach has been used to explore the hippocampal metabolic changes using proton magnetic resonance spectroscopy (1H MRS), involving a 7T MR scanner in combination with histochemical and immunofluorescence analysis. We found alterations in the metabolic profile, as well as remarkable histo-morphological changes such as an increase of hippocampal volume, alterations in number and morphology of astrocytes, neurons, and their processes in the selective vulnerable brain area of animals treated with a Met-enriched diet. Results of both methodologies suggest that the mild hHcy induced by Met-enriched diet alters volume, histo-morphological pattern, and metabolic profile of hippocampal brain area, which might eventually endorse the neurodegenerative processes.

## 1. Introduction

Several nutritional factors can influence the risk of development of neurodegenerative diseases and their rate of progression. Therefore, there is a growing interest in understanding how diet as a modifiable lifestyle factor could mitigate the risk of these diseases [1,2].

Methionine (Met) is an essential amino acid present in foods and regularly consumed within the Western diet [2,3,4,5,6]. The impact of a high and moderate Met diet on different tissues had been discussed in many studies [2,3,4]. Protein intake rich in Met content or dysregulation of Met metabolism within the “Met-homocysteine” cycle can lead to the elevation of homocysteine (Hcy) in circulating plasma. Hcy is a sulphur-containing amino acid produced by metabolic conversion of Met to cysteine. Its elevated level in plasma, hyperhomocysteinemia (hHcy), is one of the known risk factors for cardio- and cerebrovascular disorders. Clinical relevance of elevated total Hcy level in plasma has been proven by many studies in different tissues [7,8]. Remarkably, development of the neurodegenerative disorders, such as progressive atherosclerosis and acute ischemic stroke, cognitive impairment, dementia, or Alzheimer’s disease (AD), is also associated with hHcy [3,4,6], but the exact mechanism of its involvement in affecting neuronal tissue is not yet elucidated. In addition, S-adenosyl homocysteine (SAH), an intermediate of metabolic conversion of Hcy, was shown to be a metabolic sensor controlling methylation-regulated signalling within the Met-Hcy cycle. As a consequence, it potentially initiates the DNA and protein hypomethylation with an impact on diverse tissue disturbances, including neuronal cells [9]. Likewise, the selective cytosolic and nuclear Hcy clearance is essential for cellular genetic protection.

Our previous works documented that subcutaneous administration of Hcy in rats led to the development of mild hHcy [10,11,12]. This hHcy condition resulted in the disintegration of neuronal tissue in the cerebral cortex as well as the hippocampus. Moreover, Tóthová et al. [12] have shown that hHcy on the same rat model also triggers remarkable epigenetic changes with the impairment in histone acetylation, likely within the context of hHcy-initiated DNA hypomethylation in vulnerable brain areas [9].

In this paper, we used a novel methodological approach to detect the potential impact of a Met-enriched diet on alterations of metabolic ratios in the Met-Hcy cycle. Changes have been detected by the combination of in vivo proton magnetic resonance spectroscopy (1H MRS) and magnetic resonance imaging (MRI), as well as by the histo-morphological analysis. In vivo 1H MRS and MRI present a non-invasive, powerful, and precise tool that can detect imbalances in the metabolic ratio, as well as the volumetric discrepancies of organs, and are commonly used in clinical studies [13,14]. Alterations in the one carbon metabolism may exacerbate the toxic potential of Hcy and its metabolites and affect the “methylation index” with impact to DNA methylation reactions and gene regulation [15]. The model of moderate Met diet-induced hHcy was used to bring more light into the plausible pathomechanism of neurodegeneration induced by these conditions. We demonstrate here that the combination of both methodologies reveals Met-enriched diet-induced neurodegeneration processes that are associated with the alterations in metabolic ratio and morphological changes in the *cornu ammonis 1* (CA1) hippocampal area in rats.

## 2. Results

### 2.1. Determination of Plasma Hcy

Determination of plasma Hcy in animals has shown that total plasma Hcy levels in animals with 28 days of Met-enriched diet (MDG) was significantly elevated when compared to the male control (C) Wistar rats (7.15 ± 0.42 μmol/L, *n* = 5) and reached 11.38 ± 4.8 μmol/L (*n* = 5).

### 2.2. 1H MRS Analysis

Absolute concentrations of 1H MRS metabolite in the hippocampus of animals enrolled in this study together with statistical evaluation of differences in metabolite ratios between control and MDG animal group are shown in Table 1. We measured total N-Acetyl Aspartate (tNAA), myo-Inositol (mIns), total choline (tCho) and total creatine (tCr) containing compounds which were expressed as following ratios: tNAA/tCr, mIns/tNAA, mlns/tCr, tCho/tNAA and tCho/tCr.

Quantification of 1H MRS in the hippocampus of animals showed tNAA (an index of axonal integrity) to tCr, (tNAA/tCr), mIns/tNAA and mlns/tCr ratios in MDG group compared to the control. However, these changes in the metabolic ratio were not statistically significant. Furthermore, in the hippocampus of MDG group was a trend of increased in tCho metabolite ratios (tCho/tCr and tCho/tNAA) in contrast to the control animal group. We found decrease in the ratio in tNAA to both, tCr and mIns (tNAA/tCr, tNAA/mIns) as well as reduced mIns/tCr ratio. Nevertheless, the changes did not express statistical significance.

### 2.3. Volumetric Analysis

Based on MRI volumetric analysis, we found significant (*p* = 0.031) increment in the hippocampal volume in the MDG animal group (100.85 ± 1.82 mm^3^) compared to the control group (96.51 ± 4.78 mm^3^). The threshold of the normal tissue volume (volume threshold) was defined as the difference in mean MRI volumetric value of the hippocampus in control group and its standard deviation (SD). Thus, it was possible to define the percentage of hippocampal tissue volume change in all animals with respect to volume threshold (Table 2). Given the observed volume change in the regarded brain area, we could calculate an average hippocampal volume increased (10 ± 2 %) in the MDG animal group, with respect to the control group.

### 2.4. Histo-Morphological Changes in Rat CA1 of Hippocampus after Induced hHcy

#### 2.4.1. FluoroJade-C Staining

**Figure 1:**
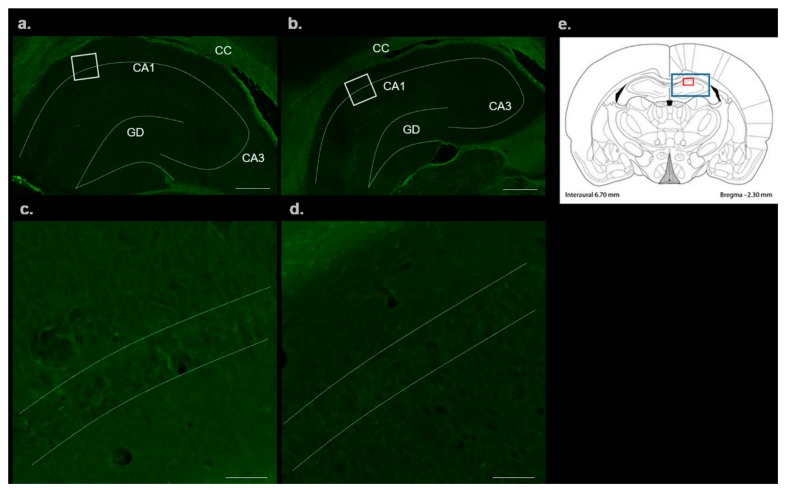
FluoroJade-C (FJC) stained rat brain sections in the *cornu ammonis 1* (CA1) region of hippocampus. Fluorescent micrographs of rat hippocampus representing control (**a**) and MDG (**b**) with a detail of corresponding group focusing on CA1 region of control (c) and MDG group (**d**). White square in the first column presents area of magnification. (**a**,**b**) Bar = 500 μm; (**c**,**d**) Bar = 50 μm; *n* = 5/group. Schematic coronal rat brain section (**e**), redrawn according to Tothova et al. [12] representing hippocampus (blue rectangle) and smaller (red rectangle) detects CA1 area of rat hippocampus.

To display the possible neurodegenerative changes induced by mild hHcy, we used FluoroJade-C (FJC) staining to detect the disintegrated neurons in the CA1 area of hippocampus. Control group was compared to the Met-enriched diet group (MDG) of animals. As shown in Figure 1a–d, we were not able to detect any FJC positive degenerating neurons in controls. In the animals with MDG, FJC staining in hippocampi was also very faint.

#### 2.4.2. Neural Nuclei and Glial Fibrillary Acidic Protein Measurement

**Figure 2:**
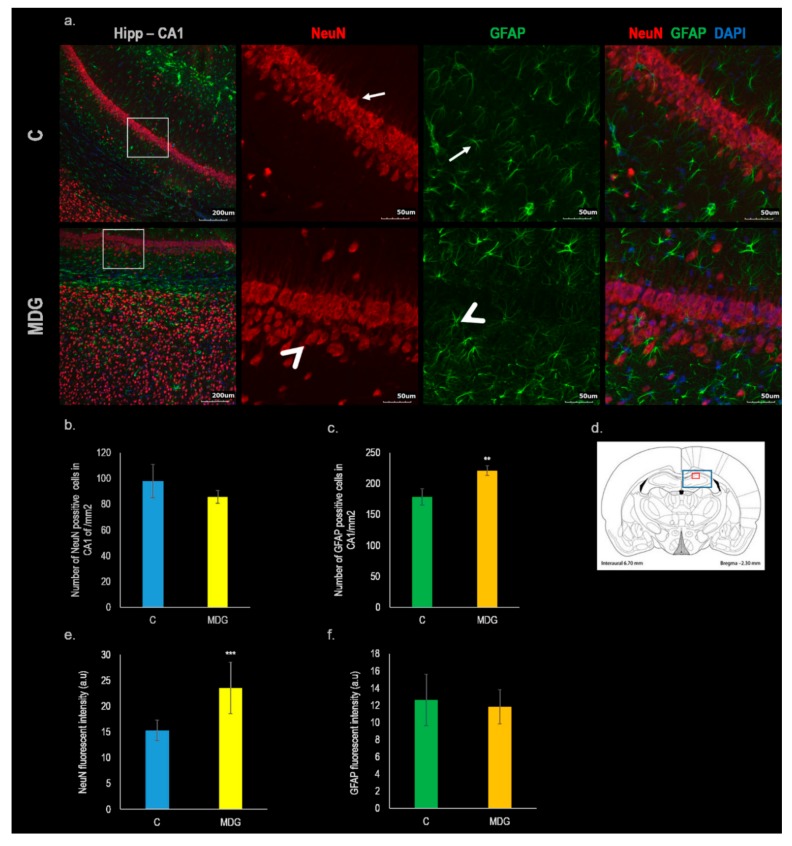
Immunofluorescent microphotographs and statistical evaluation of NeuN and GFAP in the CA1 region of hippocampus. (**a**) Fluorescent micrographs of rat hippocampus representing control and Met-enriched diet treated animals (MDG). The first column represents low magnification pictures of hippocampus in CA1 region of both experimental groups. The second column determines high magnification pictures of CA1 stained by NeuN (red). The third column indicates high magnification pictures of CA1 stained by glial fibrillary acidic protein (GFAP) (green) and the last column portrays high magnification pictures of CA1 of overlaid antibodies contra-stained by 4′,6-diamidino-2-phenylindole (DAPI; blue). The white rectangle in the first column presents the area of magnification. Arrows point to neurons and astrocytes without any impairment. Arrowheads indicate morphologically changed neurons or astrocytes. (**a**) (column Hipp-CA1) Bar = 200 μm; (**a**) (columns NeuN, GRAF and NeuN + GFAP + DAPI) = 50 μm; *n* = 5/group. Schematic coronal rat brain section (**d**), described in Figure 1. (**b**) Number of NeuN positive neurons in the CA1 region in the control group (C) and MDG group. (**c**) Number of GFAP positive astrocytes in the CA1 region. (**e**) NeuN fluorescent intensity in the CA1 region. (**f**) GFAP fluorescent intensity in the CA1 region. All results are presented as mean ± SD for *n* = 5/group, normalized to the control levels. *** *p* < 0.001 and ** *p* < 0.01 versus the control value.

Immunofluorescent labelling with neural nuclei (NeuN) and glial fibrillary acidic protein (GFAP) was used to detect potential changes in the number and/or morphological alterations of mature neurons as well as astrocytes in the hippocampal brain area. In the control group (C), NeuN antibody labelled nuclei and the cytoplasm in the majority of neuronal cell types of all regions in the adult brain including the cerebral cortex, hippocampus and cerebellum. No immunoreactivity was observed in astrocytes of CA1 subfields neither in the nuclei nor the cytoplasm. The most cytoplasmic immunopositivity was concentrated in the soma, rarely extending to a short distance into the processes (mainly axon hillock; Figure 2a). On the other hand, in the MDG group of animals (MDG; Figure 2a), we detected remarkable morphological changes in the CA1 hippocampal neurons (swelling of soma as well as nuclei; arrowhead) with no statistical differences in cell number when compared to the control (Figure 2b).

Additionally, the semiquantitative measurement of NeuN fluorescent intensity displayed statistically significant changes in the MDG group, where the intensity of fluorescent signal increased by 53.6 % (*p* < 0.001; Figure 2e) when compared to the control.

Regarding, GFAP immunolabeling in the control group, astrocytes manifest typical radially arranged thin processes. GFAP labelled astrocytes were dispersed in all layers of the hippocampus (C; Figure 2a). In the animals fed with the Met-enriched diet (MDG), we observed a remarkable increase of GFAP staining found in astrocytes located predominantly in hippocampal *stratum oriens*, *stratum radiatum*, and *stratum lacunosum*. We also found different morphology of astrocytes in the MDG group that was probably related to the changes of hHcy induced activation of astrocytes (MDG; Figure 2a). Furthermore, the number of astrocytes in MDG elevated to 123.7% (*p* < 0.001) in comparison to the control (Figure 2c).

However, the fluorescent signal of GFAP in the MDG group was not significantly changed in comparison to the control (Figure 2f).

#### 2.4.3. β-Tubulin Fluorescent Analysis

**Figure 3:**
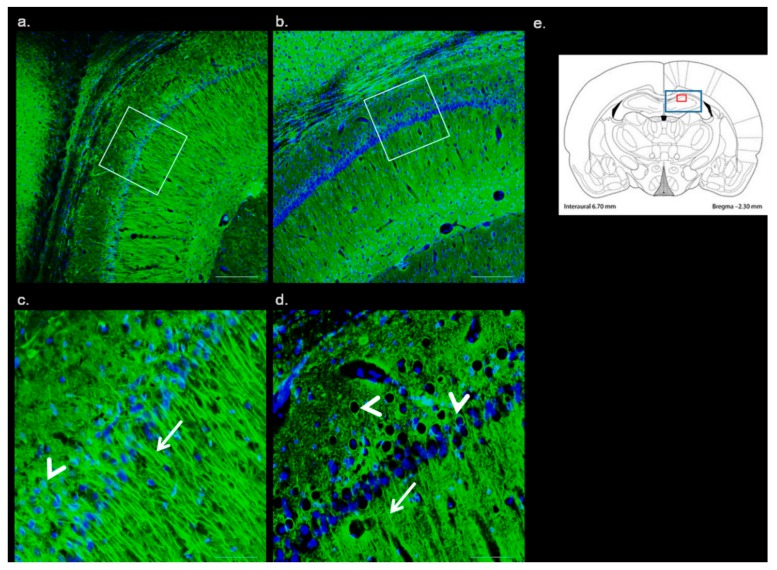
Immunofluorescent microphotographs of β-Tubulin in the CA1 region of hippocampus. Fluorescent micrographs of low magnification of rat CA1 of hippocampus representing control (**a**) and Met-enriched diet treated animals (**b**). The second row represents details of the corresponding group focusing on the CA1 region of hippocampus ((**c**): C and (**d**): MDG). Arrows indicate axons, arrowheads body on neurons. Nuclei are contra-stained by DAPI (blue). The white square in the first column presents area of magnification. Schematic coronal rat brain section (**e**), described in Figure 1. (**a**,**b**) Bar = 200 μm; (**c**,**d**) Bar = 50 μm; *n* = 5/group.

With the aim to detect changes in the neuronal architecture potentially linked to the hippocampal degenerative processes induced by Met-enriched diet, we used β-Tubulin antibody staining. Microtubules represent an important cytoskeletal structure essential for neuronal physiology with the early changes induced by neurodegeneration. Figure 3 demonstrates representative pictures from both, the control and the Met-enriched experimental groups.

In the control histologically intact tissue, β-Tubulin was predominantly located within the plasmalemma of perikarya and processes of neurons (dendrites and axons, too; Figure 3a,c). The β-Tubulin staining in the animals fed with Met-enriched diet showed changes in the thinning of axons and a decrease in expression in neuronal somas was manifested (Figure 3b,d). This might suggest an early induction of potential neurite neurodegeneration, with delayed damage to the soma. This feature resembles the phenomenon of “dying back axonopathy” [16], as a common feature of several neurodegenerative processes.

## 3. Discussion

### 3.1. Metabolic and Volumetric Changes in the Hippocampus after Met-Enriched Diet Inducing hHcy

Hippocampus is one of the most vulnerable brain regions which is linked with the selective damage of neurons induced by different noxi. High Met protein intake or dysregulation of Met metabolism can potentially lead to plasma Hcy elevation as one of the known risk factors for cardio- and cerebrovascular disorders [7,8]. HHcy is also associated with the propensity to the development of neurodegeneration linked with dementia or AD [3,4,6], but the exact mechanism of Hcy involvement is not fully clear. This paper presents a novel methodological approach using 1H MRS and MRI volumetry with the immunohistological analysis, to bring more light into the pathomechanisms associated with the Met-enriched diet–induced hHcy.

In general, it is referred that tCr has the most stable concentration among 1H MRS-detectable metabolites in the brain, and thus it is used as a reference for relative metabolite quantification [17]. However, regional and individual variability in tCr concentration is known [18]. Cr is not produced solely in the brain, and systemic renal disease as a possible cause of hHcy [8] may impact the tCr levels in the brain [13]. Glial cells manifest a four-times higher content of tCr than neurons, and thus it is considered a glial marker [13]. Remarkably, its simultaneous occurrence with the neuroaxonal marker tNAA and other glial components, tCho (mainly oligodendrocytes) and mIns (mainly astrocytes), enables us to non-invasively reflect the intracranial metabolic changes [14,18].

Here we document that Met-enriched diet is manifested by non-significant changes between the levels of all detected metabolites, but we found decrease in tNAA/tCr, mIns/tNAA, and mIns/tCr ratios with rise in tCho/tNAA and tCho/tCr. Metabolic injury resulting in axonal destruction is detectable in vivo by 1H MRS signal intensity of NAA, probably as a result of impaired metabolism. NAA is one of the most common amino acids in the brain which acts as an important organic osmolyte, and a precursor for myelin-lipids synthesis [17]. Reduced tNAA 1H MRS signal refers to neuronal/axonal/dendritic density dysfunction and/or its loss [14,18,19,20]. The tNAA levels reflect pathologic severity [21] and correlate with clinical measures in cross-sectional studies [22]. Moreover, there is only spare information on how the neuronal tissue cope with hHcy conditions at the level of metabolic changes in vivo [23]. Results of our experiments indicate that Met-enriched diet inducing hHcy is linked with the progressive metabolic disturbances, or degradation of myelinated tract in the hippocampus, which can be translated to the clinically relevant hHcy conditions.

Choline as a precursor of membrane metabolism is considered as a 1H MRS marker of membrane density, i.e., phospholipids synthesis and degradation [24]. Additionally, tCho is typically elevated during myelin sheet degradation [17,25] and an increase in tCho relative to tNAA and tCr was correlated with cerebral infarctions, ongoing gliosis, and ischemic and re/de-myelinization processes [19,25]. It was recently shown that tCho was associated with membrane turnover that was directly related to Hcy removal [24], and a link was revealed between hHcy and ceramide metabolism in AD-type neurodegeneration [17,25,26]. In our experiments, we did not find significant changes in the levels of tCho and tCr in the hippocampus, but the rise in its ratio suggests for the proposed process of hippocampal re/de-remyelination, neuroglial dyshomeostasis, and cell membrane turnover in MDG conditions.

MIns, as an important osmolyte, is synthesized primarily in glial cells representing glia proliferation or an increase in glial cell size as a result of inflammation [17]. In our experiments, we found a decreased ratio of mIns/tNAA and mlns/tCr in MDG group of animals. Decreased mIns was described early after trauma [27,28], which may reflect mIns efflux from astrocytes as a volume-regulatory strategy under conditions of oedema, or alternatively may reflect cell lysis and death [29]. By contrast, pathologically activated astrocytes with larger cell volumes tend to have an increased level of mIns [30]. A link between increased mIns and astrocyte activation was also detected in animal models of status epilepticus [31], of AD and trauma brain injury [23].

It has been noted that hHcy promotes neuronal tissue breakdown, oedema and/or cell lysis, or a shift from oxidative energy metabolism toward anaerobic glycolysis due to the mitochondrial dysfunction of [32] which significantly might affect metabolite ratio [5,6,33]. In addition, Met-Hcy cycle is connected to one carbon metabolism and editing processes in proteosynthesis and thus Hcy and its metabolites might interfere with the epigenetic control of gene expression as one of the underlying pathological factors [34]. Epigenetic dysregulation mediated by changes in DNA methylation and histone N-homocysteinylation might have an impact to the consequent metabolic disparities in hHcy conditions.

Our results clearly manifested an increase in hippocampal volume in MDG animals with the elevated level of Hcy. These conditions could be presented as a result of disruption of blood brain barrier caused by Hcy or its metabolites, as well as its direct excitotoxic effect on astrocytes [35]. It is known that hHcy can impair the endothelium’s capacity to regulate vascular tone by reduced bioavailability of NO which leads to endothelial dysfunction [15,36] and in turn affects astrocytes as we described here. Astrocytes have been shown to modulate synaptic activity and plasticity by regulating extracellular space volume as well as ion and neurotransmitter homeostasis [37]. Overall results support our volumetric analysis and suggest for the proposed cytotoxic oedema linked with the model of Met diet-induced hHcy. This is in line with documented changes in metabolite’s homeostasis such as decrease in tNAA/tCr and increase in tCho/tNAA and tCho/tCr. In addition, results of histo-morphological analysis with the ascertained changes in neurons, swelling of bodies of neuronal cells, and massive increase in number of astrocytes also support our previous data.

### 3.2. Histo-Morphological Changes in the Brain after Met Diet–Induced hHcy

It is generally believed that hippocampus comprises of different areas with diverse selective vulnerability to neurodegenerative processes [38] of which CA1 region is the most sensitive. Therefore, our histochemical and immunofluorescent analyses were focused mainly on CA1 hippocampal area to observe a plausible histo-morphological change.

Moderate Met-enriched diet for 28 days led to mild hHcy in healthy adult rats, as was also shown by similar studies [3,39]. Notably, in our experiments, this diet type was not manifested by clear positive FJC staining even in the CA1 hippocampal region. It suggests that the dose and duration of Met intake (2 g/kg within 28 days) is not linked with the clearly visible signs of neuronal disintegration in the CA1 hippocampal region. On the other hand, hHcy induced by subcutaneous administration of Hcy for 14 days was manifested by remarkable disintegration of neuronal tissues detected by FluoroJade staining in the cerebral cortex and hippocampus [10,11]. This indicates the diverse effect of hHcy induction, which is probably linked with the direct toxic impact of Hcy or its metabolites. Similar to our previous study, results in this paper clearly determined morphological changes in the neurons and elevation in glia cellularity in Met treated animals. We observed histo-morphological changes such as swelling of neuronal cell bodies linked with the dysregulation of metabolic ratio (decrease in tNAA/tCr and increase in tCho/tCr) and changes in the volumetric analysis which is consonant to the previous studies from different laboratories [40,41] and clinical studies [18,42]. This indicates that different types of the development of hHcy conditions (direct Hcy subcutaneous administration or by Met-enriched diet) could lead to the different levels of histological damage, probably influenced by Hcy itself or its toxic metabolites.

Interestingly, the number of astrocytes detected by GFAP showed a substantial increase in the MDG group. We found hypertrophic somas with elongated, thicker and branched processes of astrocytes, however, not linked with the changes in the metabolic ratio. Activation of astrocytes was referred to as a possible mal-adaptive alteration in neuronal functions [43], since astrocytes proliferation displays the early stages of the most neurodegenerative processes. Our histo-morphological results indicate for moderate astrogliosis, a well-known progressive hallmark of diseases such as AD [43,44]. Maler et al. [45] first showed in the cell culture that astrocytes exposure to D, L-homocysteine resulted in a time and dose-dependent gliotoxic effect. Jin and Brennan [46] demonstrated that sub-lethal concentrations of Hcy caused significant metabolic changes and altered mitochondrial function in primary cultures of astrocytes. Weekman et al. [35] showed in their in vitro study that after treatment with the moderate level of Hcy, astrocytes had decreased levels of several astrocytic end-feet genes at 72 h, and they manifested an increase in the matrix metalloproteinase 9 (MPP-9). Conclusively, our results indicate that hHcy induced by a Met-enriched diet in in vivo conditions initiates reactive astrogliosis in CA1 area of the rat hippocampus.

Cytoskeletal dysfunctions have been proposed as an underlying mechanism in many neurodegenerative processes [16]. It had been shown that neurotoxins, such as MPP1 [47] and 6-hydroxydopamine [48] alter microtubule dynamics, causing a decrease in length and number of microtubules and shortening of neurites. Reduction in microtubule stability also underlies the behavioral and axonal transport defects seen in Parkinson disease (PD)-associated mutations [49]. In this work, we ascertained the presence of disrupted cytoskeletal protein, reducing length or attenuation of neurites in the MDG animals. Remarkably, disruption of microtubule environment is thought to be an important parameter, which potentially can lead to the progressive neurodegenerative process. Extensive brain damage, as well as an oxidative dysbalance, has also been shown on the animal model of gestational hypermethioninemia [50].

Based on our results, we suggest that Met-enriched diet inducing hHcy represents an experimental model which produces a toxic environment with potential neuronal tissue damage linked with the changed metabolic ratio, volume disturbance, attenuated neurites and activation of astrocytes in rat hippocampus.

## 4. Materials and Methods

### 4.1. Induction of Mild hHcy by Met-Enriched Diet

Animals used in this study were carried out in accordance with guideline for Animal Care and Health of the State Veterinary and Food Department of the Slovak Republic (approval number 727/12-221 for animal experiments from 19 August 2016). Experiment was implemented according to Directive 2010/63/EU for European Parliament and of the Council on the protection of animals used for scientific purposes.

In our experiments, adult male Wistar rats (Velaz, Prague, Czech Republic), 5–6 months old and weighing 300–400 g (mean body weight of 320 g, total *n* = 18), were used. Animals were kept in air-conditioned rooms under the standard conditions, temperature (22 ± 2 °C) and 12 h day/night cycle. Food and water were available ad libitum.

Rats were subjected to Met-enriched diet throughout 28 days before the experiment. Met (L-methionine, Sigma-Aldrich, Germany) was given in drinking water at a dose 2 g/kg of animal weight per day according Xu et al. [3]. The daily volume of water intake was measured at 43.86 ± 7.25 mL for rats in both experimental groups. After this treatment, moderate hHcy was evoked in animals and determination of plasma Hcy concentration followed. At day 29, peripheral blood samples (1.5 mL) were collected from retro-orbital venous plexus of MDG and control animals, immediately cooled on ice, and centrifuged. The supernatant was collected and plasma was stored at −80 °C. The plasma Hcy levels were measured by commercially available enzymatic assay with Hcy Liquid Stable Reagent Kit (Axis-Shield Diagnostics Ltd, Dundee, Scotland) according to the instructions of manufacturer and analyzed with an automatic biochemical analyzer (Siemens ADVIA 1650, Tarrytown, NY, USA).

Animals were divided into two groups according to analysis. The first group of animals underwent histological analysis, and animals were separated into two subgroups, see 4.2 (*n* = 5/group). The second group of eight animals was analyzed by magnetic resonance (MR) twice. Before Met diet (on day 0) and in the end of Met diet (on day 28). After a particular period, the animals were sacrificed by perfusion in a mild sevoflurane anesthesia in accordance with the ethical principles. Brains were rapidly dissected from the skull and processed for future procedures.

### 4.2. Experimental Groups of Animals

The first group of rats was divided into following subgroups:

1. control animals (C, *n* = 5).
2. the animals after 28 days with Met diet (MDG, *n* = 5).

The second group consisted of eight animals that underwent the same procedures as subgroup 2, but due to different handling and analysis methods, were these animals separated into independent group.

### 4.3. FJC Staining

All of animals from group 1 (*n* = 5/subgroup) were placed in an anesthetic box and put to sleep by spontaneous inhalation of 3.5 % sevoflurane as a mixture of oxygen and nitrous oxide (33/66%). Animals were subsequently trans-cranially perfused with 0.1 mol/L phosphate-buffered saline (PBS, pH 7.4) followed by 4% paraformaldehyde in 0.1 mol/L PBS (pH 7.4). After perfusion, all animals were decapitated. The brains were removed from the skull and submerged overnight in the same fixative at 4 °C. Finally, the rat brains were placed in 30% sucrose for next 24 h at 4 °C. The rat brains were embedded with embedding medium (Killik, Bio Optica, Milano, Italy) and promptly frozen by fast cooling boost in a cryobar of Shannon Cryotome E (Thermo Scientific, Waltham, MA, USA) and sectioned at 30 μm thick sections. The sections were placed at Superfrost Plus glass (Thermo Scientific, Waltham, MA, USA). FJC was used as a marker for neurons undergoing degeneration. The sections mounted on the Superfrost Plus glasses were heated at 50 °C for at least half an hour before staining. The slides were immersed in absolute alcohol for 3 min, then 1 min in 70 % alcohol, and 1 min in distilled water. Subsequently slides were transferred to a solution of 0.06 % potassium permanganate for 15 min and rinsed in distilled water for 2 min. After 120 min in the staining solution, 3 × 1 min rinses in distilled water followed. The slides were dried at room temperature and cover slipped with Fluoromount™ Aqueous Mounting Medium (Sigma-Aldrich, St. Louis, MO, USA) according to the standard protocols.

### 4.4. Fluorescent Immunohistochemistry

Brain sections from first group of animals mounted onto Superfrost Plus glass (Thermo Scientific, Waltham, MA, USA) were permeabilized with 0.1% Triton X-100, preblocked with 10% BSA for 60 min. Primary antibody used in this work was GFAP (1:200; AB5804, Millipore, Burlington, MA, USA), which is an astrocyte-specific marker. As a marker of mature neurons, NeuN (1:100; 24307S; Cell Signaling Technology, Beverly, MA, USA) rabbit antibody was applied. For morphological determination of neuronal cytoskeleton β3 Tubulin mouse monoclonal antibody (1:50; sc-5274; Santa Cruz Technology, Dallas, TX, USA) was utilized as well.

The tissue sections were incubated with O/N at 4 °C using solution of primary antibodies diluted in the 0.1% Triton X-100 (Cell Signaling Technology, Beverly, MA, USA) enriched by 10% BSA. Immunofluorescent detection was accomplished using Alexa Fluor 488 goat-anti-mouse IgG (A11001, 1:100, Life Technologies, Carlsbad, CA, USA) conjugated secondary antibody for GFAP and β Tubulin, Alexa Fluor 594 goat-anti-rabbit IgG (A11012, 1:100, Life Technologies)-conjugated secondary antibody for NeuN. Sections were mounted with DAPI Fluoromount-G^®^ medium (CA 0100-20, SouthernBiotech, Birmingham, AL, USA) according to standard protocols. No immunoreactivity was detected in the absence of the primary antibody. The slides were examined by a confocal laser scanning microscope, Olympus FluoView FV10i (Olympus, Tokyo, Japan) in the CA1 of hippocampus. Cells were counted in a double-blind manner by two observers on three random microscopic fields. The objective of 10x with zoom up to 40x magnification equipped with filters for FITC (fluorescein isothiocyanate for Fluoro-Jade C; excitation: 495 nm; emission: 519 nm), Alexa Fluor 488 (excitation: 499 nm; emission: 520 nm), and Alexa Fluor 594 (excitation: 590 nm; emission: 618 nm) was utilized. The image capture was performed with Olympus Fluoview FV10-ASW software, version 02.01 (Olympus, Tokyo, Japan), Quick Photo Micro software, version 2.3 (Promicra, Prague, CR) and further processed in Adobe Photoshop CS3 Extended, version 10.0 for Windows (Adobe Systems, San Jose, CA, USA).

### 4.5. In Vivo MR-Examination

For in vivo MR examination, we used a 7 T Bruker BioSpec small animal MR scanner (Bruker BioSpin MRI, Ettlingen, Germany). The 1H radio-frequency resonator (Bruker BioSpin MRI, Ettlingen, Germany) was used for RF transmission. For signal reception in brain areas the 4-elements 1H surface array coil was applied (Bruker BioSpin MRI, Ettlingen, Germany).

The second group of experimental animals, control (*n* = 8) followed by Met diet as MDG group (*n* = 8) were anesthetized with sevoflurane (4% sevoflurane in medical O_2_ for induction of anesthesia and then 2–4% sevoflurane for anesthesia maintenance) and stabilized with tooth holder and nose mask in a dedicated water heated bed (Bruker BioSpin MRI, Ettlingen, Germany). Body temperature and respiratory rate were monitored during the scanning procedure.

To ensure similar head positioning, two-dimensional T_1_-weighted reference images were acquired within 12.8 s. For precise volume of interest (VOI) localization, the T_2_-weighted MRI in coronal, sagittal, and transversal planes were obtained with the two-dimensional turbo spin echo (rapid acquisition with relaxation enhancement; RARE) sequence and the following parameters: TR (repetition time)/TE (echo time) = 2680/40 ms, 23 slices with 0.5 mm thickness and 0.3 mm gap, 2 averages, RARE factor = 10, FOV (field of view) = 35 × 35 mm^2^, image size = 256 × 256, resolution = 0.137 × 0.137 × 0.5 mm^3^, and total acquisition = 2 min 14 s. Additionally, for volumetry purposes, the T_2_-weighted two-dimensional turbo RARE MRI of the whole brain in coronal plane was measured with 25 slices (0.5 mm thickness and 0 mm gap).

**Figure 4:**
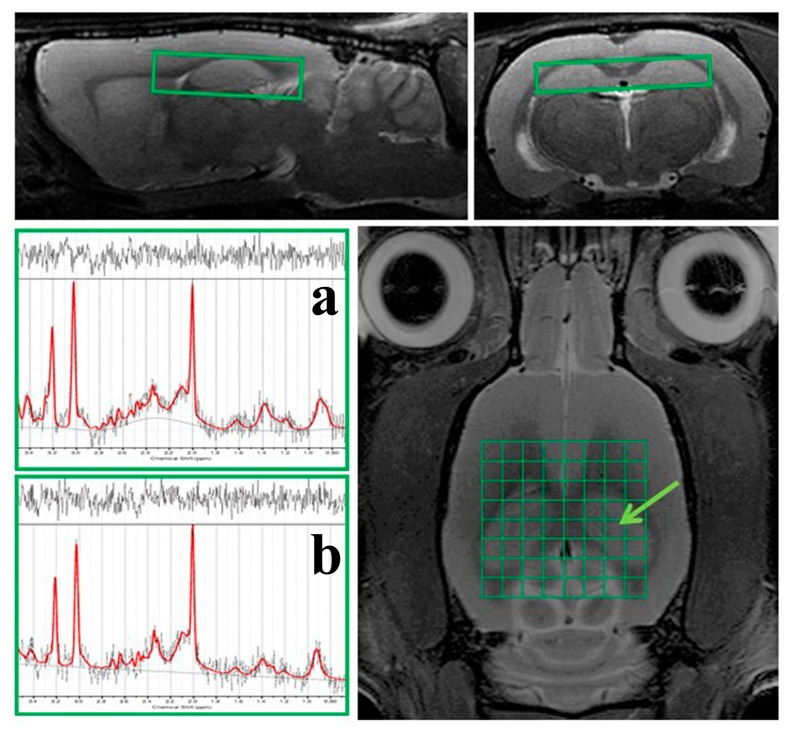
Representative picture of in vivo 1H MRS in the hippocampus of the rat brain. On the morphological T_2_-weighted MR images is shown the position of the CSI grid with real voxel size of 2.75 × 2.75 × 2 mm^3^ covering the hippocampus (green grid and boxes on MRI) of the rat brain. There are also displayed representative in vivo 1H MRS-spectra from the selected voxel (green arrow) in the rat hippocampus from control (**a**) and MDG groups (**b**) evaluated in LCModel software (version 6.3-1K; S. Provencher, Oakville, ON, Canada).

For identify magnetic field (B_0_) inhomogeneity across the brain, B_0_ phase map was obtained prior to 1H MRS. Subsequently all spectroscopic and shimming volumes were manually placed in the selected brain regions, outside of B_0_ distortions visible on the B_0_ map. Spectroscopic data from the hippocampus (*gyrus dentatus* (GD), CA1-3) were obtained in one acquisition using the chemical shift imaging (CSI) method (Figure 4). The 2D CSI measurement was performed within 16 min with PRESS (point resolved spectroscopy) pulse sequence; 8 × 8 voxel matrix and 8 × 10 × 2 mm^3^ nominal voxel size (2.75 × 2.75 × 2 mm^3^ real voxel size); 22 × 22 mm^2^ FOV; TR/TE = 1500/20 ms; 36 averages and 6 kHz acquisition bandwidth. Eddy currents and B_0_ drift compensations as well as OVS (outer volume suppression) and VAPOR (variable pulse power and optimized relaxation delays) suppression were initiated. Linear and second order shims were automatically adjusted with the cuboid shim volume. The average linewidths of water peak was 14.8 ± 1.9 Hz.

During spectroscopic data evaluation one representative voxel from the hippocampus (Figure 4) was selected. All 1H MRS data curve fitting were performed by LCModel software (version 6.3-1K; S. Provencher, Oakville, ON, Canada). Subsequently, the metabolite levels of tNAA; (N-acetyl-aspartate, N-acetyl-aspartyl-glutamate), mIns, tCho (phosphatidylcholine, glycerophosphatidylcholine, acetylcholine, choline) and tCr (creatine, phosphocreatine) containing compounds were expressed as following ratios: tNAA/tCr, mIns/tNAA, mIns/tCr, tCho/tNAA and tCho/tCr.

#### MRI Volumetric Analysis

**Figure 5:**
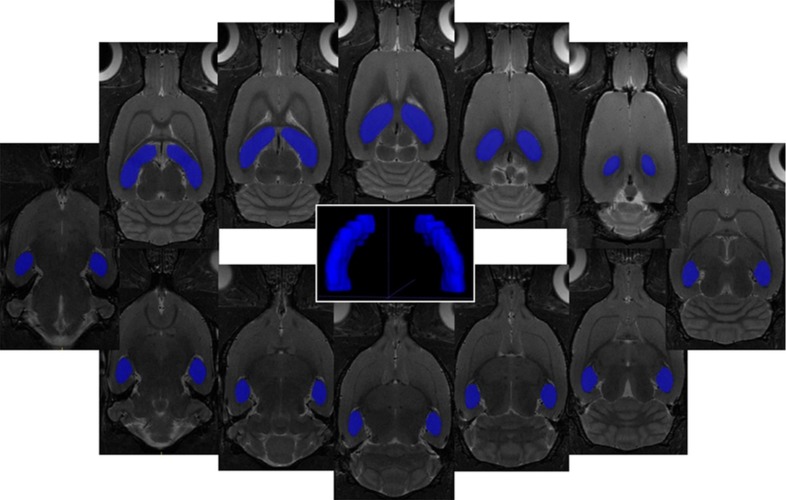
Representative pictures of in vivo MRI volumetry of the hippocampus of the rat brain. On 12 consecutive coronal T_2_-weighted MRI (resolution 0.137 × 0.137 × 0.5 mm^3^) is displayed representative ROIs covering the hippocampus of the rat brain together with 3D visualization of target areas plotted by ITK-SNAP software (Version 3.4.0, US National Institutes of Health, Philadelphia, PA, USA).

The MRI volumetric analysis of the hippocampus was performed in ITK-SNAP (Version 3.4.0, US National Institutes of Health, University of Philadelphia, Philadelphia, PA, USA) software on 12 consecutive coronal T_2_-weighted MRI slices (Figure 5). After manual target region overlaying, the selected brain tissue volume was calculated automatically. Furthermore, we calculated the hippocampal tissue normal volume threshold as the volumetric mean value calculated in the control animal group minus its standard deviation. Thus, was possible to define the percentage of hippocampal tissue volume change in all animals throughout the experiment with respect to normal volume threshold.

### 4.6. Image Analysis

Images of coronal brain sections from all experimental subgroups of first group were exported in tiff format and evaluated using ImageJ software (NIH, Bethesda, MD, USA). First RGB channels were converted to 8bit gray scale images. Threshold levels were adjusted from 13 pixels (min) to 255 pixels (max). Particle analysis was completed based upon size restrictions of 0 cm^2^-infinity leaving morphology unspecified. A total of 88 fields of view were analyzed. The numbers of NeuN+ cells (red fluorescent cytoplasm and processes), and GFAP+ cells (green fluorescent cytoplasm and processes) were directly counted in the in sections of hippocampal CA1 for each staining, respectively (2–3 sections per animal). The sampling grid size was set up to: 0.6 × 0.6 mm for each area. All counts are expressed as the total number of labelled cells per mm^2^.

### 4.7. Statistical Analysis

Data obtained from image analysis of brain sections were analyzed using GraphPad Prism software, version 6.01 for Windows (La Jolla, CA, USA). The data were analyzed using one-way and two-way analysis of variance (ANOVA). Statistical analysis for spectroscopic and MRI volumetric data was performed using the SPSS software package (Version 15.0; Chicago, IL, USA). To analyze the difference in hippocampal volumes and metabolite ratios between animal groups independent-samples, a two-tailed t-test was performed.

## 5. Conclusions

In conclusion, our study combines 1H MRS, MRI and histological analyses as a novel approach and reveals considerable cerebral metabolic dysregulation on the level of cerebral metabolites, as well as an acceleration of the hippocampal neurodegeneration in rats subjected to mild hHcy induced by Met-enriched diet. Our results also provide insight on how metabolic disturbances are translated to the histo-morphological patterns suggesting for neurodegenerative pathology in the selectively vulnerable hippocampal area. Apparently, prevention of the risk factors, participating in the Met-Hcy cycle might have an important prophylactic implication for neurodegenerative disorders, dementia and stroke. Evidence of a possible causal link between Met-enriched diet, hHcy, and neurodegenerative processes will extend the knowledge available not only at the level of basal research, but may also contribute to the better understanding in susceptibility of modifiable risk factors (hHcy) in clinical settings. Finally, the combination of a multiparametric MRI approach with histological analysis can further elaborate our understanding of complex processes linked with the metabolic neurodegenerative disorders.

## 6. Limitations

One limitation of this study was in the actual measurement of metabolites, because their level could fluctuate in chronic conditions due to occurrence of cytotoxic oedema (tissue water), which could result in a lack of correlation between 1H MRI and histological data [50]. Furthermore, different vulnerability to acute or chronic insults in the hippocampal areas [38] could also influence the actual metabolic ratio. In line of this, Zhuo et al. [33] noted that different approaches could trigger diverse downstream mechanisms by the distinct hHcy induction. Data accumulated so far indicate that the diet-induced hHcy is also affected by the strain difference established in rodents [39].

## Figures and Tables

**Table 1:** Proton magnetic resonance spectroscopy (1H MRS) in the hippocampus of rats. Relative concentrations (mean ± SD) of 1H MRS metabolite ratios (tNAA/tCr, mIns/tNAA, mIns/tCr, tCho/tNAA, tCho/tCr) evaluated in the hippocampus for control (C) and Met-enriched diet (MDG) animal group. Using independent-samples two-tailed *t*-tests (SPSS software, version 15.0; Chicago, IL, USA) were obtained *p*-values expressing statistical differences in metabolite ratios between groups.

Group Ratio	C */n = 8/* mean ± SD	MDG */n = 8/* mean ± SD	C/MDG
tNAA/tCr	1.071 ± 0.224	1.012 ± 0.121	0.612
mIns/tNAA	0.494 ± 0.235	0.466 ± 0.206	0.827
mIns/tCr	0.497 ± 0.187	0.461 ± 0.187	0.739
tCho/tNAA	0.181 ± 0.044	0.190 ± 0.035	0.717
tCho/tCr	0.188 ± 0.027	0.192 ± 0.039	0.838
tCho/tCr	0.188 ± 0.027	0.192 ± 0.039	0.838

**Table 2:** Magnetic resonance imaging (MRI) volumetry in the hippocampus of rats. Tissue volumetric values (mean ± SD; gray background) of the hippocampus for control (C; *n* = 8) followed by Met diet (MDG; *n* = 8) treated animals. Using independent-samples two-tailed t-tests (SPSS software, version 15.0; Chicago, IL, USA), were not obtained *p*-values expressing statistical differences in metabolite ratios between the groups (yellow background). Displayed is hippocampal normal volume threshold (C and percentages of tissue volume change; mean ± SD; green background) and hippocampal volume change in the MDG group with respect to normal volume threshold in the hippocampus.

HIPPOCAMPUS		
volume change mean ± SD tissue volume mean ± SD	C (volume threshold) 91.7 mm^3^	MDG 10 ± 2 %
C 96.51 ± 4.78 mm^3^	-	independent sample 2-tailed t-test
MDG 100.85 ± 1.82 mm^3^	0.031	-

“-“ means the statistical significance between C/C and MDG/MDG.

## Abbreviations

AD	Alzheimer´s disease
CA1	Cornu ammonis 1
FJC	FluoroJade-C
GFAP	Glial fibrillary acidic protein
Hcy	Homocysteine
hHcy	Hyperhomocysteinemia
1H MRS	Proton magnetic resonance spectroscopy
MDG	Methionine diet group
Met	Methionine
mIns	Myo-Inositol
MRI	Magnetic resonance imaging
NeuN	Neural nuclei
tCho	Total choline
tCr	Total creatinine
tNAA	Total N-ademosyl-aspartate
